# “Covid madness”

**DOI:** 10.1192/j.eurpsy.2021.797

**Published:** 2021-08-13

**Authors:** C. Capella Meseguer, E. Rodríguez Vázquez, J. Gonçalves Cerejeira, I. Santos Carrasco, L. García García

**Affiliations:** 1 Psiquiatría, HCUV, Valladolid, Spain; 2 Psiquiatría, Hospital Clínico Universitario de Valladolid, Valladolid, Spain

**Keywords:** covid 19, SECONDARY EFFECT, Induced Mania

## Abstract

**Introduction:**

We present the case of a man who, after receiving treatment for Covid-19 pneumonia, suffers a manic episode induced by medication.

**Objectives:**

This case is chosen to present as an example of a psychiatric illness derived from Covid-19, in this case secondary to its treatment.

**Methods:**

We present a manic episode induced by covid medication.

**Results:**

Complementary examinations are carried out in which organic pathology is ruled out, being diagnosed of a manifest episode in probable relation to the treatments used and hospital admission is decided due to the impossibility of home management. Neuroleptic and anxiolytic treatment was started at low doses with a good response, and he was discharged with complete recovery and critiqued of the episode.

**Conclusions:**

It is estimated that between 20-40% of patients with COVID infection have presented neuropsychiatric symptoms. Mania secondary to treatment was reported in 13 (0 · 7%) of 1744 patients with coranavirus included in a study (1). The Spanish Pharmacivigilance System of Medicines for Human Use (SEFV-H) closely monitors the suspected adverse reactions reported with drugs considered potential therapeutic strategies for SARS-CoV-2 infection. 327 cases of adverse reactions have been reported, of which 18 cases include 28 thermal cases of suspected psychiatric adverse reactions. The interest of this case lies in the fact that it illustrates a psychiatric disorder derived from Covid-19, in this case secondary to the treatment used, which causes a manifest episode with a typical evolution in this type of case with rapid improvement at low doses of antipsychotics.

